# Supplementary material for: Electrochemistry-assisted selective butadiene hydrogenation with water
Source: Nat Commun. 2023 Apr 13;14:2106. doi: 10.1038/s41467-023-37708-1 (PMC10102003; doi:10.1038/s41467-023-37708-1)
Supplement: Supplementary file 1 — Supplementary Information [file 41467_2023_37708_MOESM1_ESM.pdf]

Electronic Supplementary Information

Electrochemistry-assisted selective butadiene hydrogenation with water

Yong-Qing Yan[1†], Ya Chen[1†], Zhao Wang[1,2*], Li-Hua Chen[1], Hao-Lin Tang[1,2], Bao-Lian Su[1,3*]

[1] State Key Laboratory of Advanced Technology for Materials Synthesis and Processing, School of Materials Science and Engineering, Wuhan University of Technology, 122, Luoshi Road, Wuhan, 430070, China.

[2] Foshan Xianhu Laboratory of the Advanced Energy Science and Technology, Guangdong Laboratory, Xianhu Hydrogen Valley, Foshan 528200, P. R. China

[3] Laboratory of Inorganic Materials Chemistry (CMI), University of Namur, B-5000 Namur, Belgium

† These authors contributed equally: Yong-Qing Yan, Ya Chen,

Correspondence and requests for materials should be addressed to Z.W (email: zhao.wang@whut.edu.cn) or to B-L. S (email: bao-lian.su@unamur.be)

1. Supplementary Figures 3
2. Supplementary Table 18
3. Supplementary Discussion 1..19
4. Supplementary Discussion 2..27

1. Supplementary Figures

Supplementary Fig. 1 | (a) Pictures of the electrochemistry-assisted selective hydrogenation system and **(b)** the gas-fee fixed-bed reactor; **(c)** Proposed reaction route for the electrochemistry-assisted selective hydrogenation;

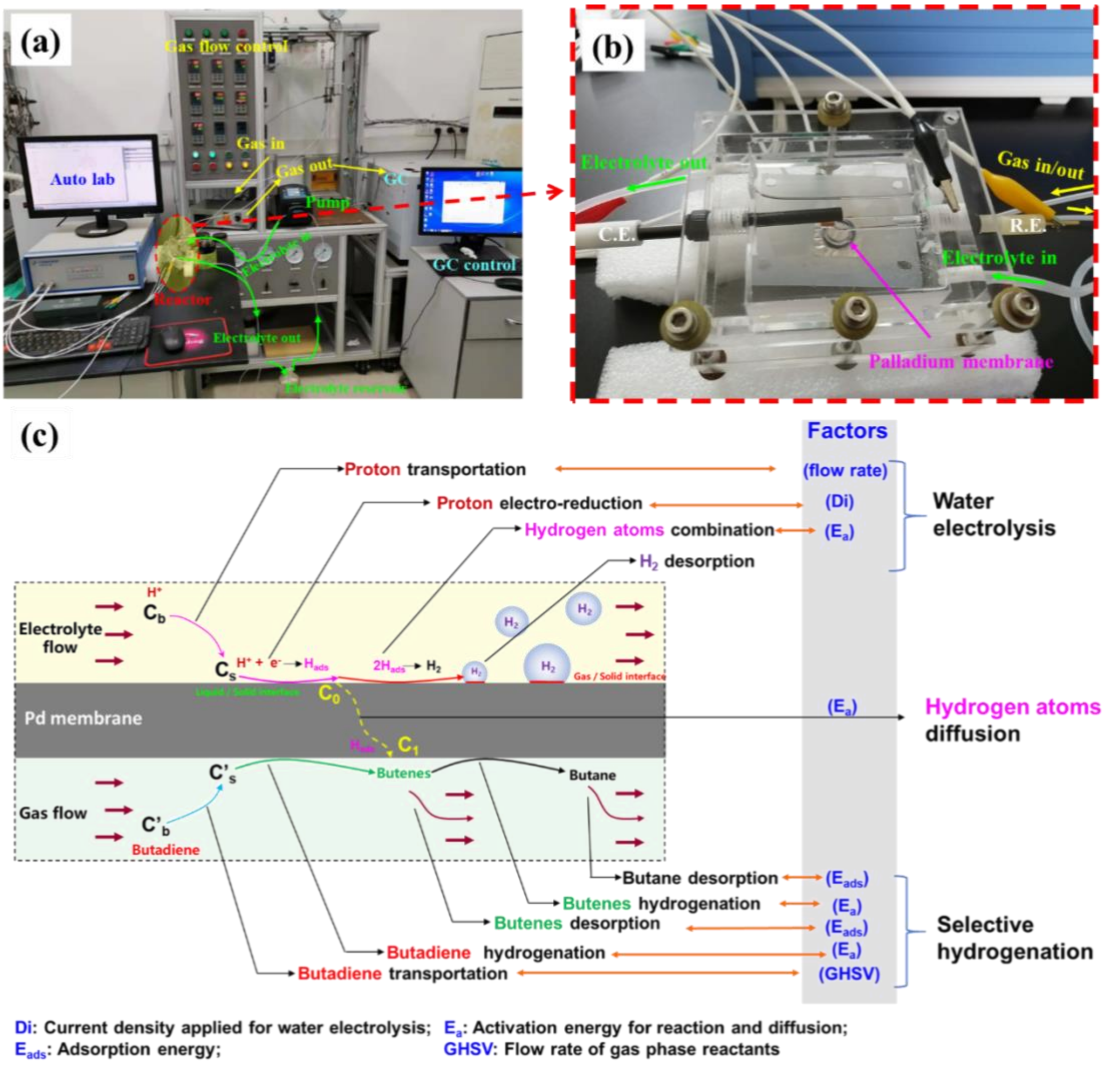

Supplementary Fig. 1c shows the whole reaction route of electrochemistry assisted hydrogenation. In detail, it contains three sections: water electrolysis, hydrogen atoms diffusion and selective hydrogenation reaction. As for water electrolysis, protons firstly diffuse onto Pd surface, then they are reduced by the electrons offered by the employed current density on Pd surface, with formation of adsorbed hydrogen atoms. The formed hydrogen atoms combine to hydrogen molecular (H_2), with the growth of

H_2 bubble on Pd membrane. This section was mainly affected by both proton transportation from electrolyte to the surface of Pd membrane (i.e., flow rate of electrolyte), the current density employed on Pd membrane (D_i) and the activation energy of hydrogen atoms combination. As for hydrogen atoms diffusion in Pd membrane, it was mainly decided by the activation energy of diffusion. Moreover, in selective butadiene hydrogenation section, butadiene is firstly hydrogenated to butenes, then to butane. The factors for this reaction include: the butadiene transportation (i.e., GHSV), the activation energy of butadiene hydrogenation (Ea), the adsorption energy of butenes (E_{ads}), the activation energy of butenes hydrogenation (E_a) and the adsorption of butane (E_{ads}). Thus, to offer a full map of the catalytic reaction, the electrolyte flow in liquid phase cell is controlled by a pump for water electrolysis, and the gas flow of butadiene reactants is adjusted by mass flowmeter for hydrogenation reaction in the gas phase cell.

Supplementary Fig. 2 | XRD patterns of as-received commercial palladium membrane.

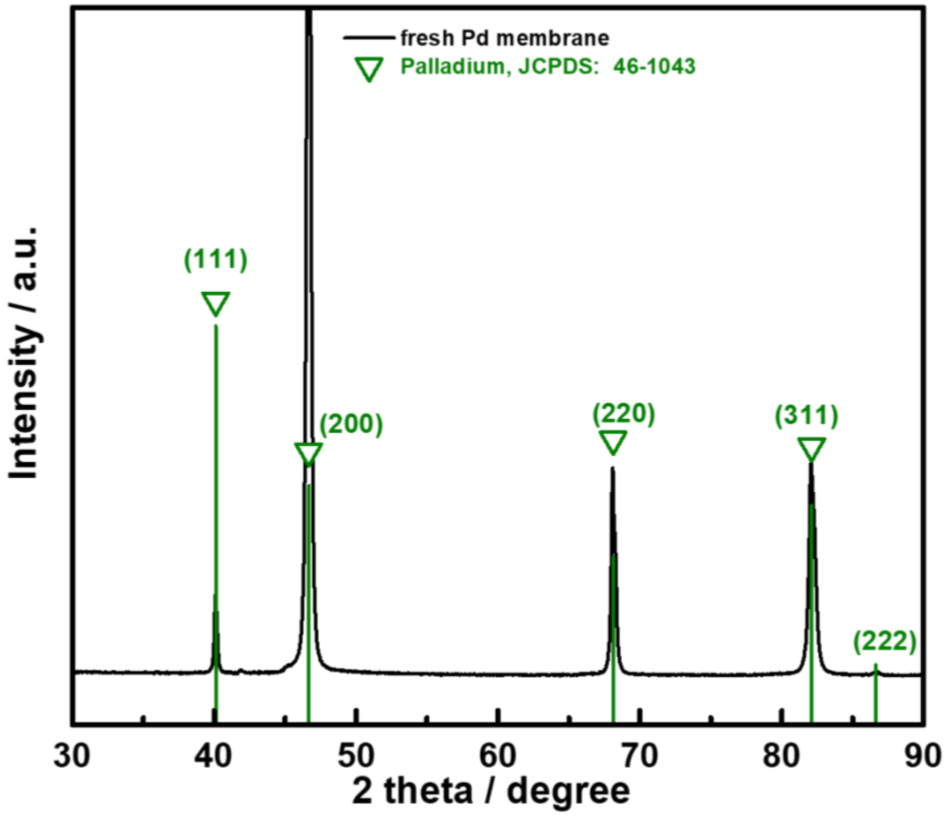

Supplementary Fig. 3 | (a) AFM and **(b)** SEM images of the fresh palladium membrane and **(c, d)** SEM of palladium membrane after stability test**.**

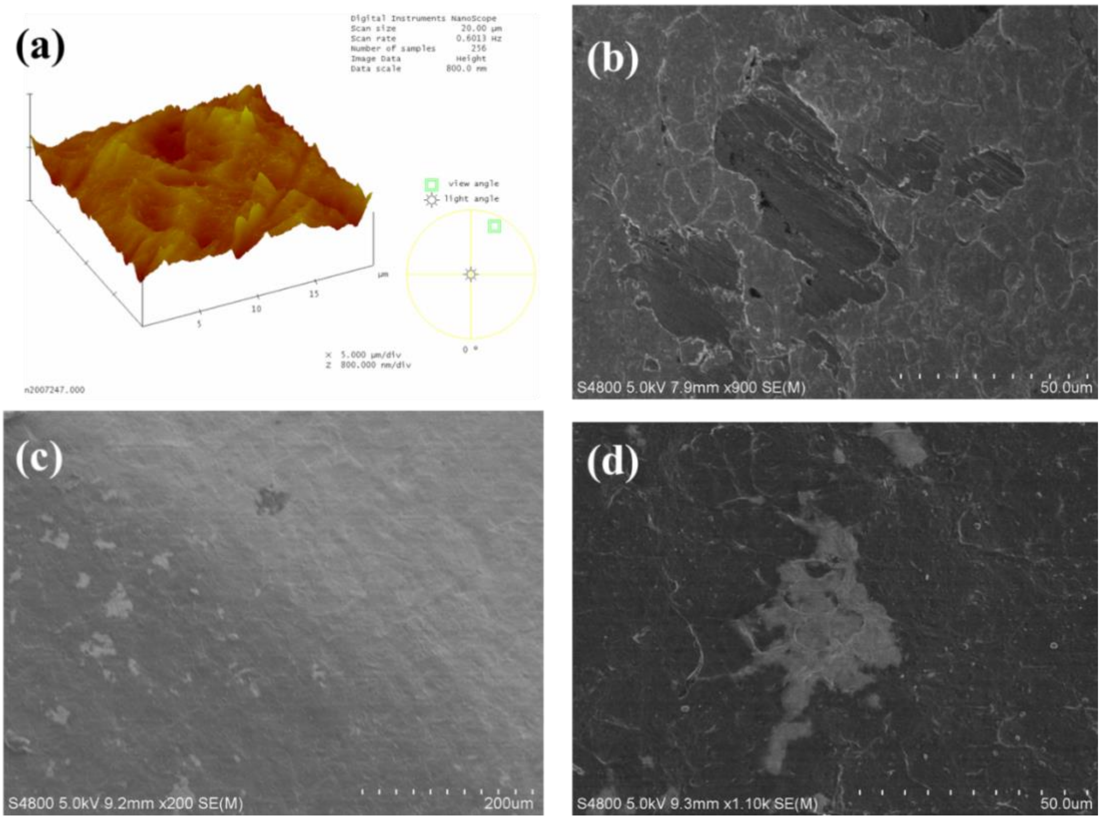

Supplementary Fig. 3 shows that the fresh palladium membrane has a rough surface, while the surface becomes slight flat after 330 h of catalytic reaction.

Supplementary Fig. 4 | Catalytic performance of using alkynes as reactant. The digital curve of GC data of acetylene (3000ppm) semi-hydrogenation in an excess of propene (3%) under different current densities at the GHSV of ~5308 h^{-1};

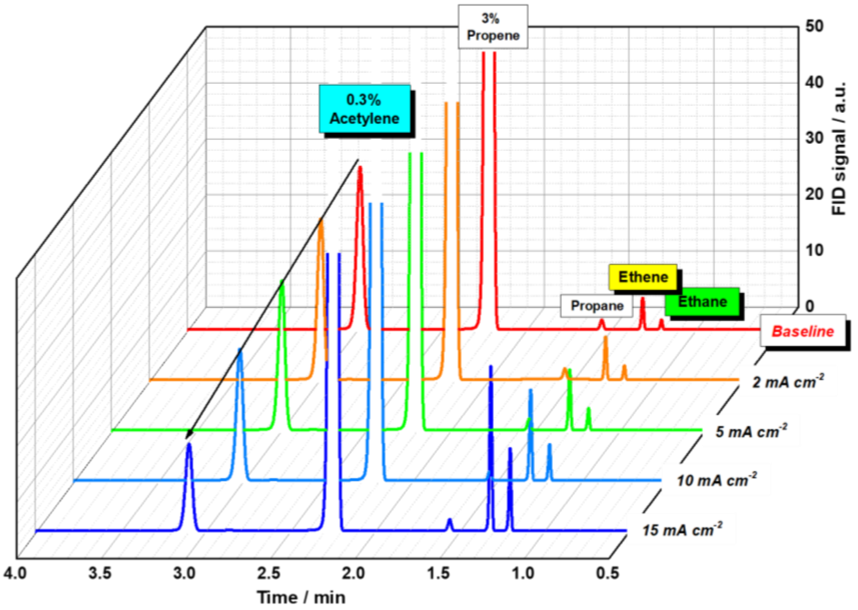

Supplementary Fig. 5 | Electrochemical property of the palladium membrane. **(a)** The cyclic voltammetry scan in 0.1M H_2SO_4 electrolyte, with a rate of 10 mA s^{-1}; **(b)** The linear sweep voltammetry scan in 0.1M H_2SO_4 electrolyte, with a rate of 5 mA s^{-1}; **(c)** The cyclic voltammetry scan in 0.2M KOH electrolyte, with a rate of 10 mA s^{-1}; **(d)** The linear sweep voltammetry scan in 0.2M KOH electrolyte, with a rate of 5 mA s^{-1};

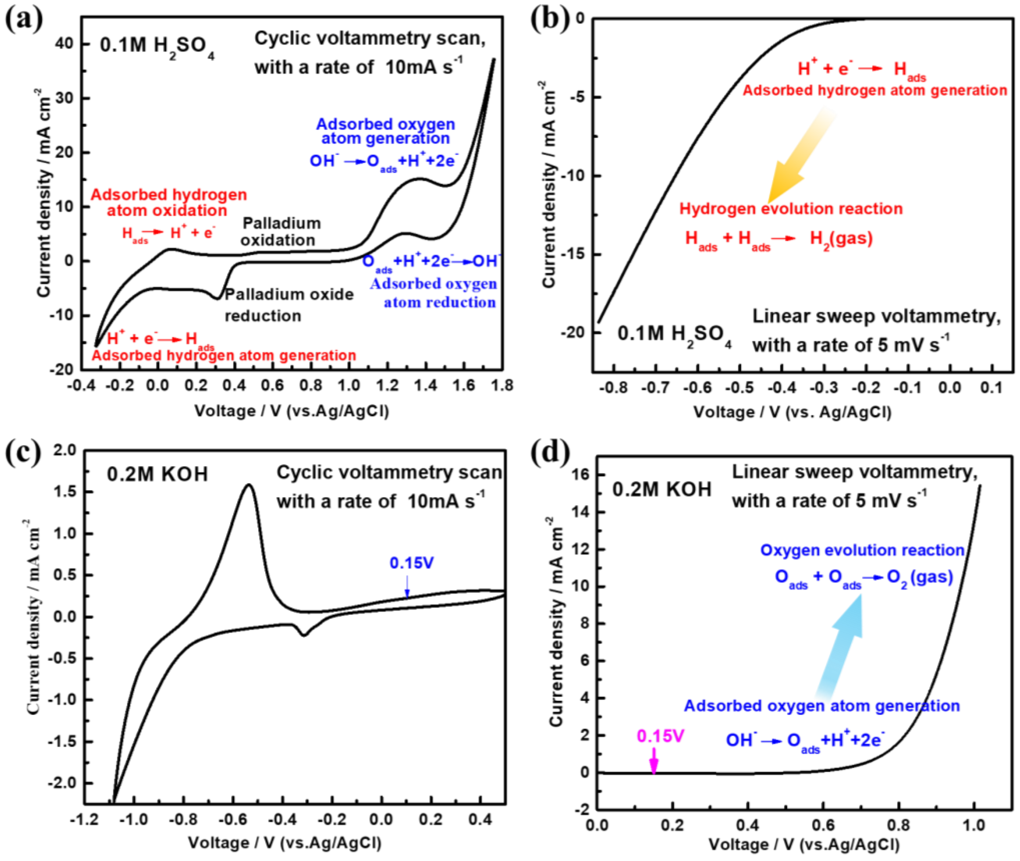

Supplementary Fig. 6 | (a) Scheme of hydrogen atom diffusion on palladium; **(b)** The energy evolution during hydrogen atom horizontal diffusion over Pd (111) surface with corresponded diffusion route; **(c)** The surface energy of Pd(111) with H coverage at 0.33 and 0.37ML; **(d)** The energy evolution during hydrogen atom vertical diffusion into Pd lattice, with corresponded intermediate structures (IS: initial state; MS: middle state; FS, final state);

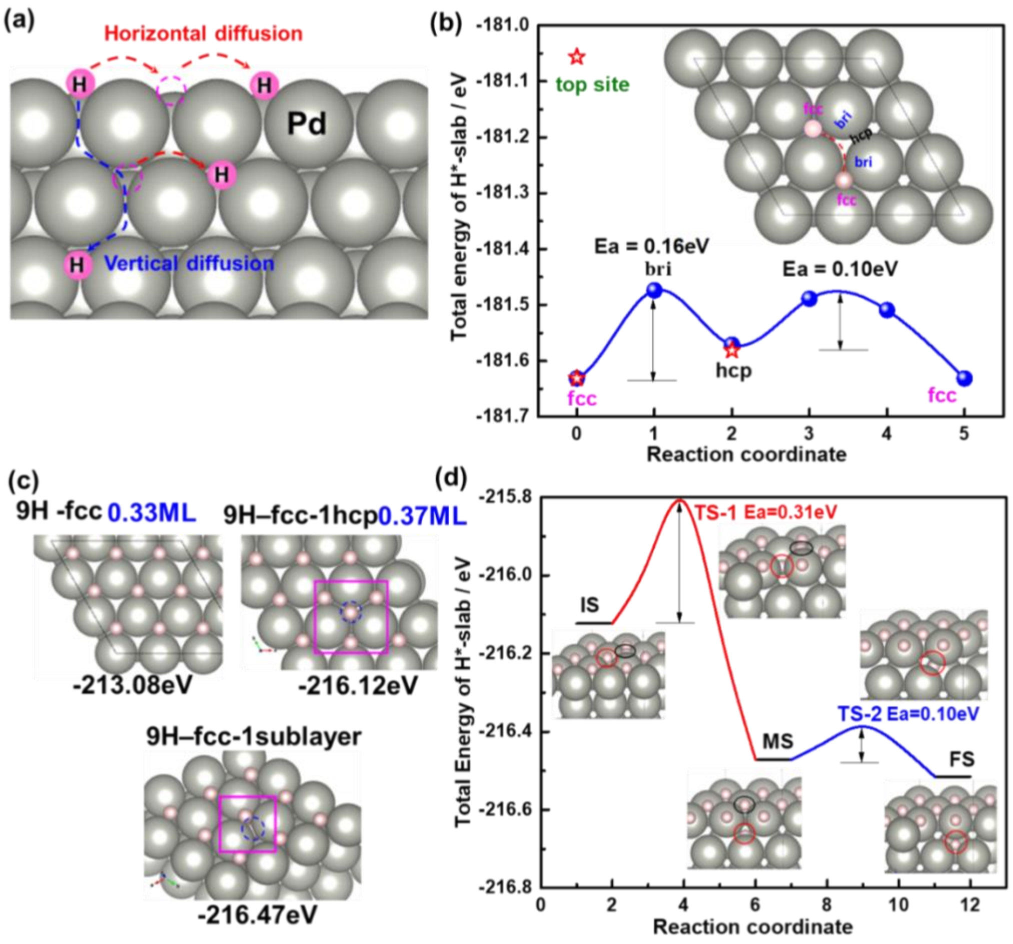

Supplementary Fig. 7 | (a) XRD patterns of palladium membrane before and after electrochemistry assisted selective butadiene hydrogenation reaction with **(b)** corresponded palladium-based crystal structures.

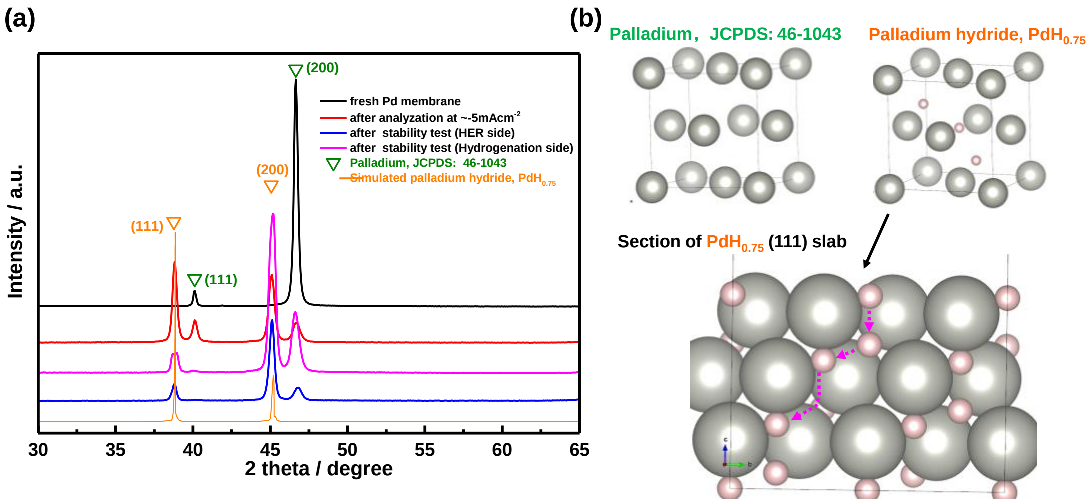

Supplementary Fig. 8 | The effect of reactants feeding method on the catalytic performance; (a) Butadiene and electrolyte aqueous for water electrolysis input into two separated cells; **(b)** Premixed butadiene/H_2**; (c)** Butadiene and H_2 input into two separated cells;

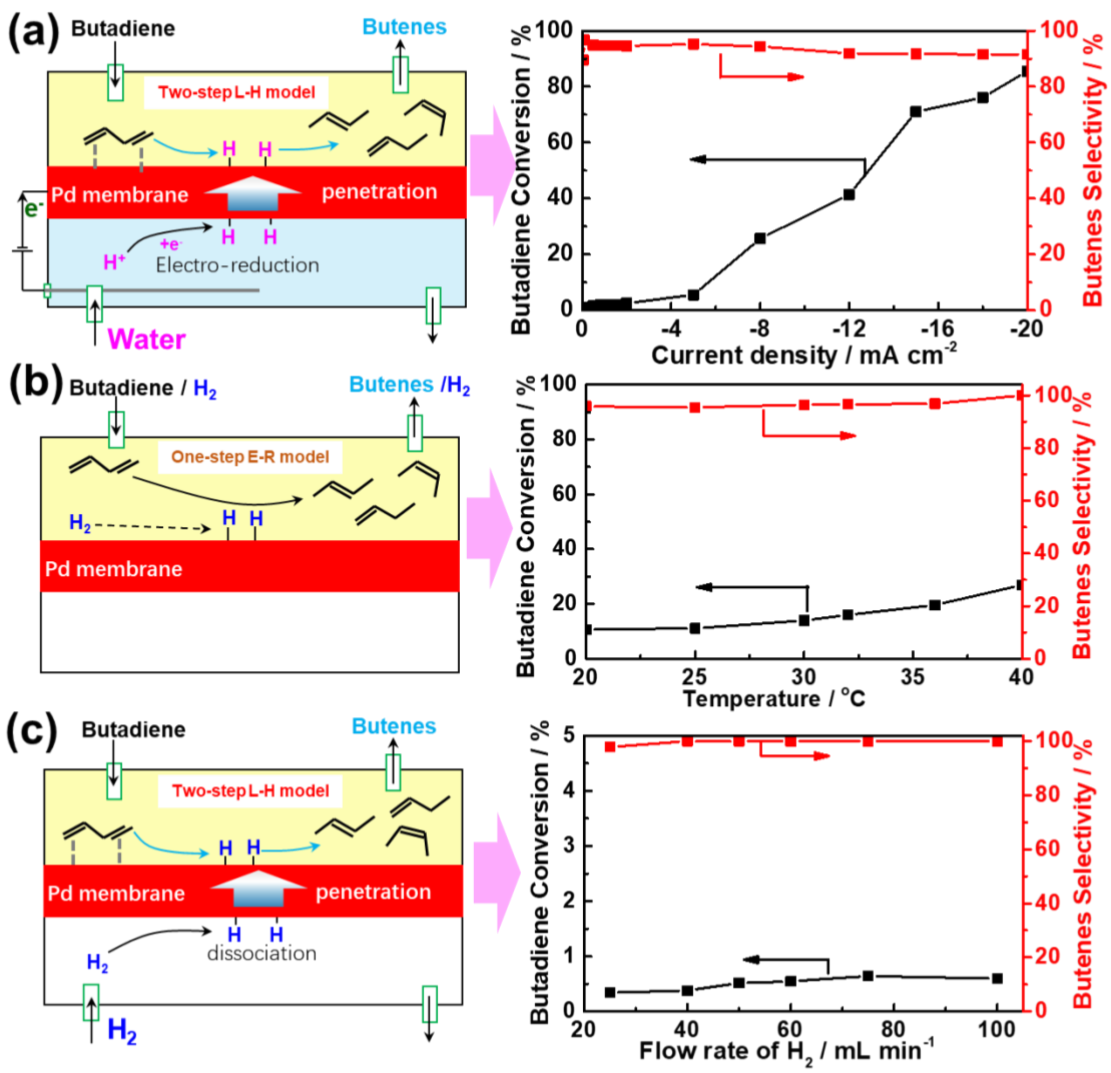

Supplementary Fig. 9 | Ab initio theoretical calculation with density functional theory for the butadiene selective hydrogenation reaction. (a) The energies of different intermediate compounds over Pd(111) during butadiene hydrogenation reaction, with corresponded structures; **(b)** Activation energy of butadiene hydrogenation over Pd(111)**. (c)** The combination energy of two separated hydrogen atoms on Pd (111) for the formation of H_2, with corresponded structures. **(d)** Activation energy of butadiene hydrogenation over Pd(335), with corresponded structures of transition state (TS);

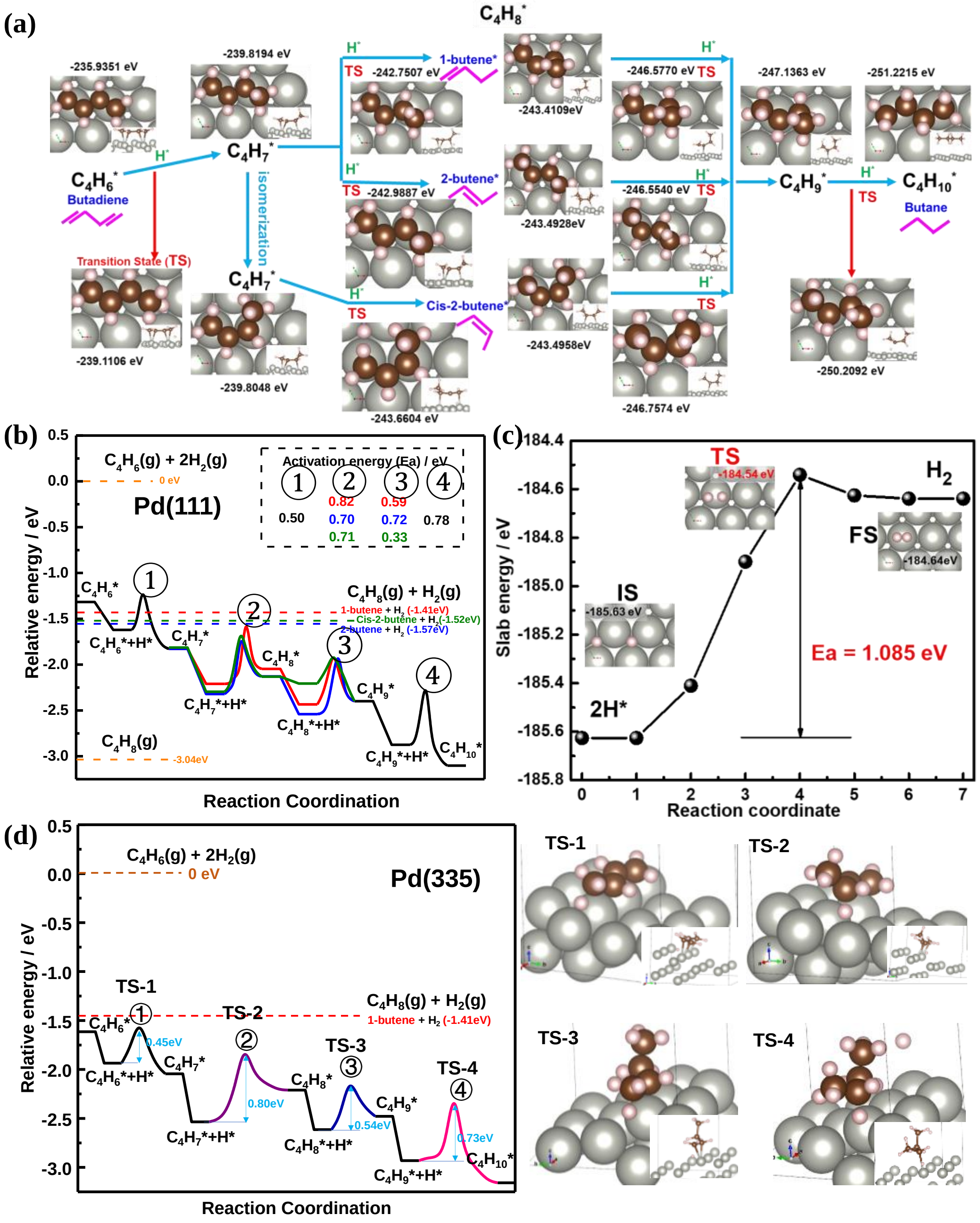

Density functional theory (DFT) calculation was firstly performed to identify the activation energy (Ea) of butadiene hydrogenation to butenes (i.e., 1-butene, 2-butene

and cis-2-butene) and butane over the most active Pd (111) surface[1], in Pd crystal. **Supplementary Fig. 9a** shows the structures and corresponded energies of the all reactants, including intermediates and translation states, during butadiene hydrogenation reaction. We also identified an isomerization reaction in the C_4H_7* species, which results in different types of butene molecular. **Supplementary Data. 9b** shows the energy evolution during butadiene hydrogenation reaction, with corresponded activation energies for all reaction steps. The rate determining step during butadiene hydrogenation was identified to be the formation of 1-butene, which has the highest Ea of 0.82eV. It is slightly higher than the empirical activation energy (i.e., 0.80 eV[2]) and indicates that 1, 3-butadiene could spontaneously react with H_{ads} on the palladium membrane surface at room temperature. Moreover, DFT calculation also shows that the adsorption energies of the reactants and products on Pd (111) surface vary as follows: butadiene (-1.32eV) < H_{ads} (-0.64eV) < butenes (-0.63eV ~ -0.55eV, depending on butene structure) < butane (-0.07eV) (in **Fig. 4a**). This means that the formed butenes on palladium surface can easily escape and be replaced by butadiene molecules and H_a owing to their lower adsorption energy, resulting into a high catalytic selectivity to butenes during butadiene selective hydrogenation reaction. **Supplementary Data. 9c** shows the activation energy is 1.08eV for the formation of hydrogen molecular from two adsorbed hydrogen atoms.

Supplementary Fig. 10 | (a) XRD and **(b)** TEM of the commercial 10wt% Pd/Al_2O_3 catalyst.

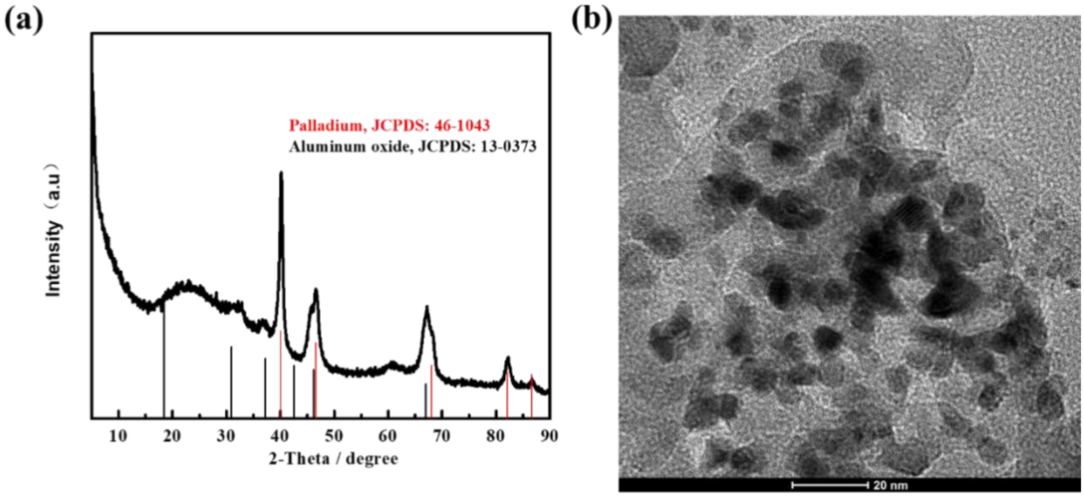

Supplementary Fig. 10 shows that two phases were identified by XRD, with metallic palladium (JCPDS:46-1043) and alumina oxide (JCPDS:13-0373). Moreover, the particle size of supported Pd is around 10 nm shown in TEM images.

Supplementary Fig. 11 | the evolution of voltage and current as function of stream on time during the stability test of electrochemistry assisted butadiene selective hydrogenation;

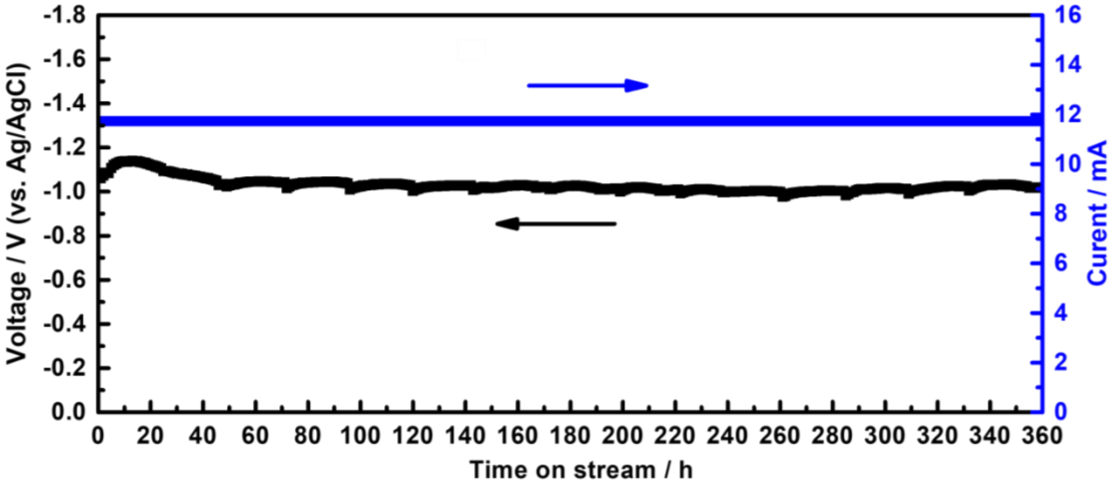

Supplementary Fig. 11 shows a low and stable voltage during the stability test of butadiene hydrogenation reaction under a constant current (i.e., -15mA cm^{-2}). The average voltage is around 1.03V (vs. Ag/AgCl), and the current is 11.78 mA.

Supplementary Fig. 12 | In-situ analyzation on the composition of outlet gas after catalytic reaction. The digital curve of GC-MS data during catalytic reaction. **(a)** A scheme of analyzation; **(b)** FID analysis; **(c)** TCD analysis; **(d)** MS analysis;

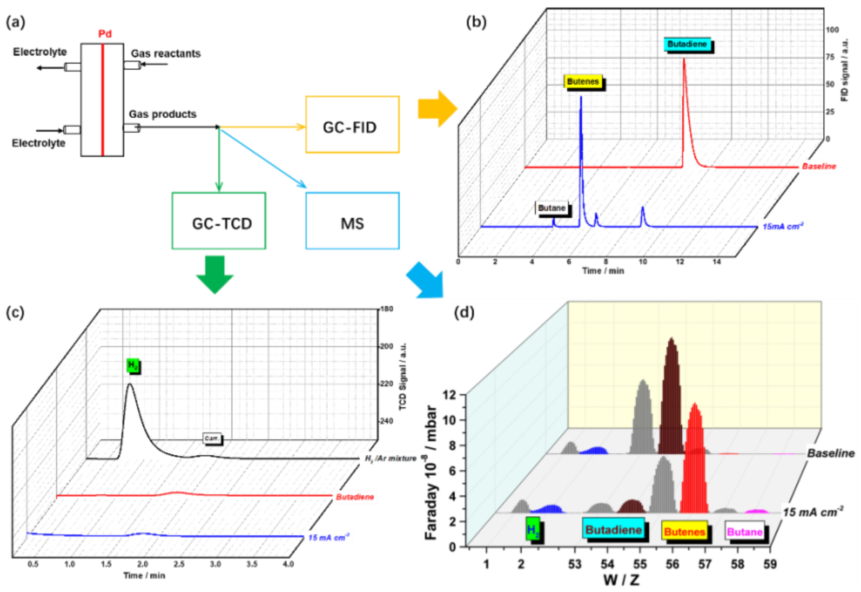

Supplementary Fig. 13 | Catalytic performance of using alkenes as reactants. The digital curve of GC data during catalytic reaction with various alkenes feedstocks under different current densities at the GHSV of ~5308h^{-1}. **(a)** 0.6% of 2-butene in helium; **(b)** 3% of propene in helium;

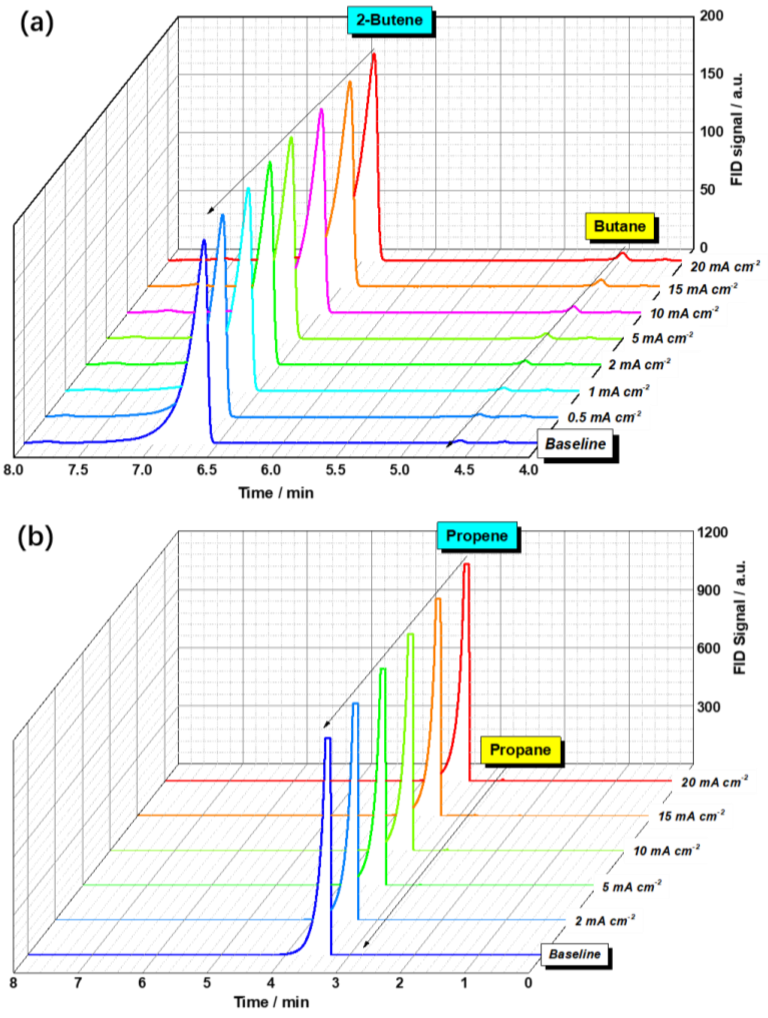

2. Supplementary Table

Supplementary Table 1. The calculation of the energy consuming in different studies. (The power of furnace was minimized at 1.2KW for the energy consuming calculation of all the thermo-catalytic reaction**)**

Catalyst	Reaction condition	Energy consuming	Reference
Pd membrane	Reaction temperature: RT<br>Hydrogen: 0<br>Time on stream: 360 h	0.003 $Wh/mL_{butadiene}$	This work
Pt1-Cu/Al_2O_3	Reaction temperature: 160°C<br>Hydrogen: 16%<br>Time on stream: 12h	20 $Wh/mL_{butadiene}$	Nat. Commun., 2015, 6, 8550-8555
0.2Pt/m-Al_2O_3	Reaction temperature: 200 °C<br>Hydrogen: 16%<br>Time on stream: 24h	50 $Wh/mL_{butadiene}$	Nat Commun., 2017, 8:16100.
Pd1/graphene	Reaction temperature: 50 °C<br>Hydrogen: 4.7%<br>Time on stream: 50h	44.3$Wh/mL_{butadiene}$	J. Am. Chem. Soc. 2015, 137, 33, 10484–10487
Pd-polyphenylene sulfide	Reaction temperature: 100 °C<br>Hydrogen:0.9%<br>Time on stream: 200h	14705.9$Wh/mL_{acetylene}$	Sci. Adv., 2020, 6 (28): eabb7369
$Ni_3ZnC_{0.7}$/o-CNT	Reaction temperature: 200 °C<br>Hydrogen:4.5%<br>Time on stream: 12h	67.3$Wh/mL_{acetylene}$	Nat Commun., 2020, 11, 3324
Cu1/nanodiamond-graphene	Reaction temperature: 200 °C<br>Hydrogen: 10%<br>Time on stream: 60h	200 $Wh/mL_{acetylene}$	Nat Commun., 2019 10 (1):4431.
Pd_1-Nanodiamond-Graphene	Reaction temperature: 180 °C<br>Hydrogen: 10%<br>Time on stream: 30h	66.7$Wh/mL_{acetylene}$	J. Am. Chem. Soc. 2018, 140, 41, 13142–13146
Al13Fe4	Reaction temperature: 200 °C<br>Hydrogen: 5%<br>Time on stream: 20h	158.7$Wh/mL_{acetylene}$	Nature Mater., 2012, 11, 690–693
Na-Ni@CHA	Reaction temperature: 90 °C<br>Hydrogen: 16%<br>Time on stream:20h	>40$Wh/mL_{acetylene}$	J. Am. Chem. Soc. 2019, 141, 25, 9920–9927
AgPd0.01/SiO_2	Reaction temperature: 160 °C<br>H2: 20%<br>Time on stream: 24h	95.2$Wh/mL_{acetylene}$	ACS Catal. 2015, 5, 6, 3717–3725

3. Supplementary Discussion 1

The hydrogen atom penetration in Pd for hydrogenation

In this electrochemical-assisted selective hydrogenation process, H_a crossing over palladium membrane from liquid-phase cell to gas-phase cell plays a core role as it was introduced in the Supplementary Fig. 1c. Further exploration about the effect of hydrogen atom penetration on Pd on the catalytic performance was discussed in this part.

The Supplementary Fig. 5 shows the basic electrochemical property of palladium membrane in acidic and basic electrolytes. The whole electrochemical reaction (presented in Supplementary Fig. 1a) can be observed with the applied voltage (vs. Ag/AgCl) in the range -0.4V to 1.8V in the electrolyte of 0.1M H_2SO_4. Among them, the adsorbed hydrogen atom generation and the hydrogen evolution reaction (HER) mainly happens at voltage lower than -0.1V (Supplementary Fig. 5b). Moreover, the voltage for the whole reaction has a negative shift in basic electrolyte (0.2M KOH in Supplementary Fig. 5c). Importantly, there is no adsorbed oxygen generation (i.e., OH^- electro-oxidation) and the oxygen evolution reaction (OER) at voltage of 0.15V (vs. Ag/AgCl).

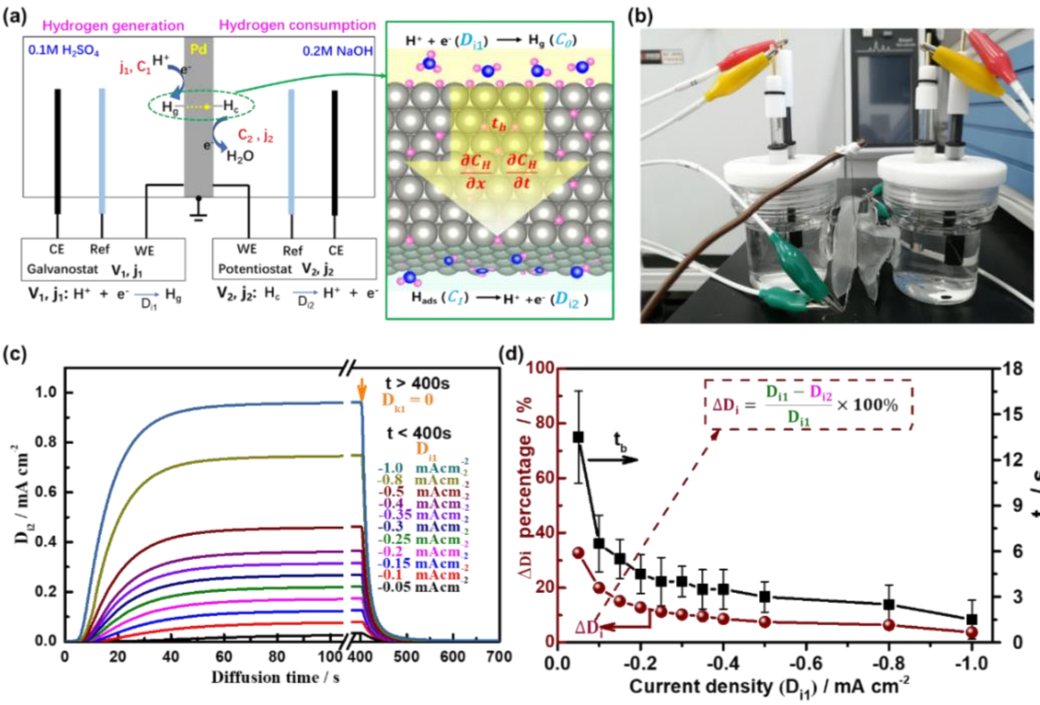

Supplementary Fig. 14 | Hydrogen atom diffusion in Pd membrane. (a) The proposed mechanism of HAET method for testing hydrogen atom diffusion; (b) Pictures of the device for the hydrogen atom electrochemical titration test; (c) Evolution of D_{i2} as a function of the diffusion time at different current densities; (d) The break through time (t_b) and the gap between D_{i2} at D_{i1} (ΔD_i) at steady state as a function of the current density (D_{i1}) for water electrolysis, with one standard (SD) error setting for break through time (t_b);

The H_a transportation behavior in Pd membrane was analyzed by hydrogen atom electrochemical titration test (HAET)[3,4], i.e., H_a generation (labeled as H_g) and consumption (labeled as H_c) separately happen on the two sides of Pd membrane by loading reduction voltage (V_1)/current (j_1) with constant of oxidation voltage (V_2 =0.15V) in two cells, sandwiched with Pd membrane as a communal working electrode (Supplementary Fig. 14a-b). The amount of H_g and H_c can be quantified by the D_i, i.e., D_{i1} and D_{i2}, respectively. Firstly, the evolution of D_{i2} as function of D_{i1} was recorded in Supplementary Fig. 14c, which shows that D_{i2} remains close to 0 for a certain time (t_b), then increases, and reaches a steady state, and finally decreases to 0 after D_{i1} is back to 0. Here, t_b is the time needed for the penetration of H_g to the Pd surface in oxidation

cell. It can be found that, in Supplementary Fig. 14d, t_b drastically decreases from above 13 s to around 6 s, then linearly decreases to lower than 1.5 s with increasing D_{i1} from -0.05 to -0.15 and finally to-1.0 mA cm^{-2}, respectively. H_a diffusion in Pd lattice obeys the Fick's first law, with the concentration gap between two sides of Pd membrane (C_0-C_1, ΔC) as the driving force[5,6]. Thus, two-stage behavior was observed on the gap (i.e., ΔD_i, directly proportional to ΔC) between the loaded D_{i1} and the D_{i2} at steady state, with a decrease from 32% to 15% and then to below 5% (Supplementary Fig. 14d). It indicates a different H_a penetration behavior in palladium, with critical D_i point at -0.15 mA cm^{-2}.

According to the Fick's first law,

$$J_H = -D\left(\frac{\partial C_H}{\partial x}\right) = \frac{dQ_H}{Adt} \quad (1)$$

In which, J_H is the diffusion flux of hydrogen atoms across palladium; D is diffusion coefficient of hydrogen atom in palladium; $\partial C_H/\partial x$_is the concentration gradient of hydrogen atom; Q_H is the amount of hydrogen atom; A is the area for diffusion, t is time. During the HAET test, the generation and consumption of hydrogen atoms in two cells are:

$$H^+ + e^- \rightarrow H^0 \quad \text{(Hydrogen atom generation) (2)}$$

$$H^0 \rightarrow H^+ + e^- \quad \text{(Hydrogen atom consumption) (3)}$$

Here, the amount of hydrogen atom is directly ratio to the number of electrons.

$$Q_H \propto Q_{e^-} \quad (4)$$

Moreover, the current density is:

$$D_i = \frac{dQ_{e^-}}{A \times dt} \quad (5)$$

As a result,

$$\frac{\Delta D_i}{\Delta t} \propto \frac{\Delta J_H}{\Delta t} \propto \frac{\Delta C_H}{\Delta t} = k \quad (6)$$

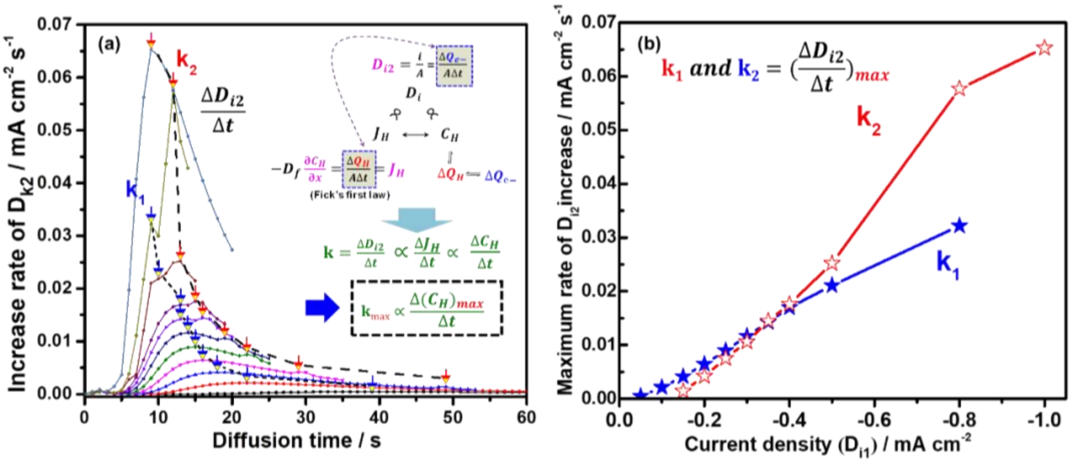

Supplementary Fig. 15 | (a) The evolution of $\Delta D_{i2}/\Delta t$ as a function of the diffusion time; (b) The maximum of $\Delta D_{i2}/\Delta t$ (k_1 and k_2) as a function of the current density (D_{i1}) for water electrolysis;

The dynamic distribution of H_a concentration (i.e., $\Delta C_H/\Delta t$) during diffusion process was further expressed as by drawing the change rate of D_{i2} as a function of time (i.e., $\Delta D_{i2}/\Delta t$) in Supplementary Fig. 15a, which shows two peaks (k_1 and k_2).These two peaks of $\Delta D_{i2}/\Delta t$ indicates the presence of two separated rich-H_{ads} layers in the Pd membrane during H_{ads} diffusion (e.g., D_{i2}) reaching its steady state. Since D_{i1} is controlled as a constant (i.e., surface H_{ads} concentration is a constant) for the recording of D_{i2} evolution during the test, the two separated rich-H_{ads} layers might indicate two different hydrogen penetration behaviors for hydrogen atom in Pd lattice. The initial point of k_2 appears at -0.15mA cm^{-2} (Supplementary Fig. 15b), which agrees with the critical point of current density (D_{i1}) for t_b and ΔD_i.

Using H/Pd (111) slab as the mode, DFT calculation recognized that H_a can horizontally diffuse sur Pd lattice surface and vertically diffuse into Pd lattice

(Supplementary Fig. 6a), with the activation energy for horizontal diffusion (labeled as E_a(H)) and vertical diffusion (labeled as E_a(V)) are 0.16 eV and 0.31 eV, respectively. Specifically, DFT calculation was performed on the energy evolution of H_{ads}/Pd(111) slab with different hydrogen atom coverages (see Method) to explore the diffusion behavior of H_{ads} in/on Pd lattice. Firstly, Supplementary Fig. 6b show that H_{ads} prefers to stay at the hollow on face centered cubic-packed (fcc) site of the Pd (111) surface, rather than at the hollow site on hexagonal closest packed (hcp) site and at atop (top) site on Pd atom, as attested by the lowest energy (-181.63 eV). Moreover, the horizontal diffusion route of hydrogen atom on Pd(111) was from fcc site to hcp site, and finally to the neighbored fcc site, with an activation energy for H_{ads} jumps to the neighbored hollow site of hcp site is -0.16 eV (Supplementary Fig. 6b).

During the exploration on the relationship between hydrogen atom coverage with the system energy, a critical hydrogen coverage (i.e., 0.33 monolayer, ML) was found. Hydrogen atom tends to stay at sub-surface in palladium lattice once all surficial fcc sites are occupied (i.e., 0.33ML) due to the relative lower energy (Supplementary Fig. 6c). Based on this result, the mode of the hydrogen vertical diffusion route was built and calculated to follow the surface-fcc site, to the sub-surface-hcp site then stabilized at the neighbored fcc site, with activation energy 0.31 eV and ~0.10 eV, respectively (Supplementary Fig. 6d). It can be found that, as for hydrogen atom, horizontal hoping on the surface is more energy favorable than that of vertical penetration into palladium lattice.

Under very low D_i (i.e., below -0.15mA cm^{-2}), H_a was slowly produced by the

electro-reduction of proton and gradually cover palladium surface. H_a prefers to firstly occupy the interstitial hole of face centered cubic (fcc), then the hexagonal closest-packed (hcp) hole of Pd (111) owing to the relative low adsorption energy (E_{ads}, Supplementary Fig. 6b). As for the DFT calculation, this reaction route could be simulated by using the slab model with unfixed (i.e., x,y and z axis) H_{ads} on Pd surface in Supplementary Fig. 16a. As for this type of unfixed H_{ads} slab, during the DFT calculation, it always offers the lowest surface energy at the final state due the energy self-optimization arithmetic in this calculation method. The E_{ads} evolution as a function of H coverage summarized in Supplementary Fig. 16b shows the changes in adsorption energy (unfixed-ΔE_{ads}) for H_a occupying the fcc hole and the hcp hole are 0.02 and 0.09eV, which are lower than the E_a(H) and the E_a(V). The penetration of hydrogen atom occurs when the surface fcc sites are occupied.

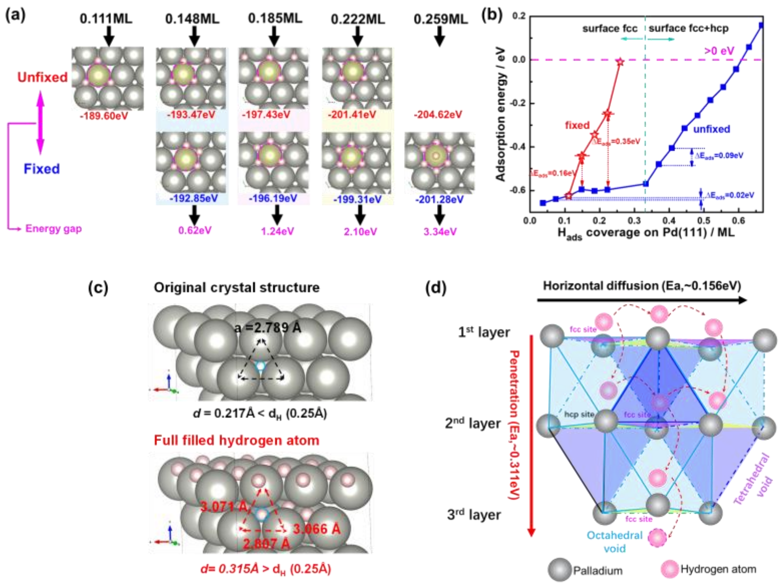

Supplementary Fig. 16 | (a) Comparison in the H_{ads}/Pd(111) slab energy between the H_{ads} with unfixed and fixed axis; (b) Adsorption energy obtained from DFT calculation as a function of H_{ads} coverage on Pd(111); (c) The size of micropore connecting octahedron void and tetrahedral void in Pd lattice; (d) Proposed vertical diffusion route for H_{ads} in Pd lattice;

However, at high D_i with polycrystalline surface of Pd membrane, H_a was rapidly formed and aggregated around the defect sites of palladium surface (i.e., corner, edge)[7,8], resulting in an increase of E_{ads} at H_a-rich Pd site. The inhomogeneous proton electro-reaction promotes aggregated hydrogen atoms around the catalytic active site on Pd membrane, resulting in high surface energy. However, this high surface energy state is an intermediate state during the reaction, and it will rapidly disappear by promoting either hydrogen atoms combination to hydrogen molecular (H_2), or hydrogen atom diffusion into palladium lattice, with a decrease of the surface energy to a minimum state. Therefore, to check the energy of this intermediate reaction state, the x and y axis must be fixed to prevent the hydrogen diffusion during the energy self-optimization in DFT calculation (Supplementary Fig. 16a). Supplementary Fig. 16b shows that the ΔE_{ads} (i.e., 0.16 eV) is equal to the $E_a(H)$ with increasing H_a coverage from 0.11 to 0.15 ML, moreover, it is above the $E_a(H)$ and the $E_a(V)$ when H_a coverage changes from 0.19 to 0.22 ML. Both horizontal diffusion and vertical diffusion appears on Pd membrane, offering two H_a rich layers distribution in Pd membrane, corresponding to the D_i higher than 0.15 mA cm^{-2} in Supplementary Fig. 15a. Moreover, at H coverage above 0.26 ML, the ΔE_{ads} is 0.58 eV, which is larger than the activation energy (-0.54 eV) for H_a combination (Supplementary Fig. 16b). The hydrogen evolution reaction[9] appears in the liquid cell, with H_2 bubble formation, resulting in a decrease of the FE, e.g., specially at D_i higher than 15 mA cm^{-2}. Supplementary Fig. 16c indicates an expended crystal structure when the sub-layer of Pd was occupied by H_a. The diameter of the interstitial hole between octahedron void and tetrahedral void in Pd crystal

increases from 0.217 Å to 0.315 Å, which is larger than the diameter of hydrogen atom (0.25 Å). It creates a connected path for H_a diffusion (schemed in Supplementary Fig. 16d), and offers an enhanced H_a diffusion for catalytic reaction, i.e., the critical point of TOF and FE at -5mA cm^{-2} (Fig. 2b).

Moreover, after butadiene hydrogenation test, an additional phase was formed in the two sides of palladium membrane for water electrolysis and butadiene hydrogenation reaction, respectively. The crystal structure of the new formed phase was simulated as palladium hydride (i.e., $PdH_{0.75}$) by the VESTA software, with simulated X-ray diffraction included in Supplementary Fig. 7a. It shows the palladium hydride is also face-centered cubic (fcc) structure, with a cell parameter of 4.02 Å×4.02 Å× 4.02 Å and 90.0°×90.0°×90.0° in Supplementary Fig. 7b. Four palladium atoms and three hydrogen atoms are included in one crystal, with hydrogen atoms staying in two tetrahedral voids and one octahedral void of palladium crystal. Comparison with fresh palladium membrane (cell parameter of 3.89019Å×3.89019 Å×3.89019Å and 90.0°×90.0°×90.0° in Supplementary Fig. 7b), a swelled palladium lattice was observed due to the dissolution of hydrogen atoms. Moreover, we cleave the (111) crystal surface (in Supplementary Fig. 7b), the distribution of hydrogen atom in the cross section of Pd (111) highly agrees with the hydrogen atom diffusion route predicted by DFT calculation in Supplementary Fig. 16d.

4. Supplementary Discussion 2

The effect of reactants feeding method on the catalytic performance

Water electrolysis as the source for hydrogen permeation is important for obtaining high catalytic performance in this developed process. Supplementary Fig. 7 present the catalytic performance of our developed palladium-membrane reactor by various types of feeding. Firstly, feeding premixed butadiene/H_2 (volume ratio of 0.6:20) reactants into reactor, butadiene conversion stays below 30% at temperature around 40°C (Supplementary Fig. 7a). This low catalytic performance was mainly due to the rather low surface area (i.e., ~1 cm^2) of bulky palladium membrane for the E-R model of reaction. Moreover, using H_2 gas as the source of hydrogen permeation in Supplementary Fig. 7b at room temperature (~20°C), butadiene conversion is below 1%, which is lower than that of premixed butadiene/H_2 reactants (~10% in Supplementary Fig. 7a). Since hydrogen penetration in Pd lattice obeys the Fick's first law[5,6], the driving force for hydrogen penetration is the concentration gap (ΔC_H) of hydrogen atom along the thickness direction of Pd membrane. The difference in butadiene conversion these two ways in Supplementary Fig. 7a-b indicates a low amount of hydrogen atom penetration by using H_2 gas. Decades years ago, few explorations have been carried on the selective hydrogenation of alkynes/alkadienes in alkenes feedstocks in palladium membrane reactor by using H_2 gas for hydrogen permeation[10], however, it was almost fully abandoned in the past years due to its inferior catalytic performance with high reaction temperature[11-13], e.g., ~80% of alkadienes conversion in palladium membrane reactor at temperature above 130°C by feeding H_2[14].

However, it was surprised to observed that, with water electrolysis for hydrogen atom generation and penetration, the butadiene conversion increase from below 5% at $D_i<5$ mA cm^{-2} to above 90% at D_i > 15mA cm^{-2} at room temperature (Supplementary Fig. 7c). The further DFT calculation in Supplementary Fig. 9 indicates that the hydrogen atoms at hollow site need much larger activation energy (Ea, ~1.09 eV) for the combination to H_2 than that of penetration (Ea, ~0.31 eV in Supplementary Fig. 6d), thus the formed hydrogen atom prefer to penetration into palladium membrane. Importantly, the catalytic selectivity to butenes was almost independent from the butadiene conversion increasing and kept at above >90% in electrochemical-assisted selective hydrogenation process.

References

1 Ding, L. *et al.* Activating Edge Sites on Pd Catalysts for Selective Hydrogenation of Acetylene via Selective Ga2O3 Decoration. *ACS Catalysis* **6**, 3700-3707, doi:10.1021/acscatal.6b00702 (2016).

2 Liu, D. *et al.* Theoretical investigation of selective hydrogenation of 1,3-butadiene on Pt doping Cu nanoparticles. *Applied Surface Science* **456**, 59-68, doi:https://doi.org/10.1016/j.apsusc.2018.06.123 (2018).

3 Devanathan, M. A. V., Stachurski, Z. & Tompkins, F. C. The adsorption and diffusion of electrolytic hydrogen in palladium. *Proceedings of the Royal Society of London. Series A. Mathematical and Physical Sciences* **270**, 90-102, doi:doi:10.1098/rspa.1962.0205 (1962).

4 Gabrielli, C., Grand, P. P., Lasia, A. & Perrot, H. Investigation of hydrogen insertion in palladium using permeation transfer function techniques. *Journal of Electroanalytical Chemistry* **532**, 121-131, doi:https://doi.org/10.1016/S0022-0728(02)00949-X (2002).

5 Hara, S. *et al.* Hydrogen diffusion coefficient and mobility in palladium as a function of equilibrium pressure evaluated by permeation measurement. *Journal of Membrane Science* **421-422**, 355-360, doi:https://doi.org/10.1016/j.memsci.2012.08.002 (2012).

6 Li, Y. & Cheng, Y.-T. Hydrogen diffusion and solubility in palladium thin films. *International Journal of Hydrogen Energy* **21**, 281-291, doi:https://doi.org/10.1016/0360-3199(95)00094-1 (1996).

7 Xia, Y., Campbell, C. T., Roldan Cuenya, B. & Mavrikakis, M. Introduction: Advanced Materials and Methods for Catalysis and Electrocatalysis by Transition Metals. *Chemical Reviews* **121**, 563-566, doi:10.1021/acs.chemrev.0c01269 (2021).

8 Shi, Y. *et al.* Noble-Metal Nanocrystals with Controlled Shapes for Catalytic and Electrocatalytic Applications. *Chemical Reviews* **121**, 649-735, doi:10.1021/acs.chemrev.0c00454 (2021).

9 Lin, C., Batchelor-McAuley, C., Laborda, E. & Compton, R. G. Tafel–Volmer Electrode Reactions: The Influence of Electron-Transfer Kinetics. *The Journal of Physical Chemistry C* **119**, 22415-22424, doi:10.1021/acs.jpcc.5b08044 (2015).

10 Armor, J. N. Catalysis with permselective inorganic membranes. *Applied Catalysis* **49**, 1-25, doi:https://doi.org/10.1016/S0166-9834(00)81418-9 (1989).

11 Dittmeyer, R., Höllein, V. & Daub, K. Membrane reactors for hydrogenation and dehydrogenation processes based on supported palladium. *Journal of Molecular Catalysis A: Chemical* **173**, 135-184, doi:https://doi.org/10.1016/S1381-1169(01)00149-2 (2001).

12 Nakatsuji, H., Hada, M. & Yonezawa, T. Theoretical study on the catalytic activity of palladium for the hydrogenation of acetylene. *Surface Science* **185**, 319-342, doi:https://doi.org/10.1016/S0039-6028(87)80629-5 (1987).

13 Gryaznov, V. M., Ermilova, M. M., Gogua, L. D., Orekhova, N. V. & Morozova,

L. S. Selective hydrogenation of diene C5 hydrocarbons on Pd-Ru membrane catalysts. *Bulletin of the Academy of Sciences of the USSR, Division of chemical science* **29**, 1871-1876, doi:10.1007/BF00949646 (1980).
14 Itoh, N., Xu, W. C. & Sathe, A. M. Capability of permeate hydrogen through palladium-based membranes for acetylene hydrogenation. *Industrial & Engineering Chemistry Research* **32**, 2614-2619, doi:10.1021/ie00023a026 (1993).
